# Clinicopathological characteristics and prognostic analysis of Lauren classification in gastric adenocarcinoma in China

**DOI:** 10.1186/1479-5876-11-58

**Published:** 2013-03-06

**Authors:** Miao-zhen Qiu, Mu-yan Cai, Dong-sheng Zhang, Zhi-qiang Wang, De-shen Wang, Yu-hong Li, Rui-hua Xu

**Affiliations:** 1 State Key Laboratory of Oncology in South China, Department of Medical Oncology, Sun Yat-sen University Cancer Center, Guangzhou, 510060, China; 2 State Key Laboratory of Oncology in South China, Department of Pathology, Sun Yat-sen University Cancer Center, Guangzhou, 510060, China

**Keywords:** Gastric cancer, Lauren classification, Prognostic analysis

## Abstract

**Background:**

According to the Lauren classification, gastric adenocarcinomas are divided into diffuse and intestinal types. The causative attribution explaining the dismal prognosis of diffuse-type remains unknown.

**Methods:**

We examined the archive of 1000 patients with gastric adenocarcinomas who received radical gastrectomy in our center and assessed the effect of the Lauren classification on survival in a multivariate approach. Moreover we compared the variation of clinical features between the diffuse-type and intestinal-type and explored the contributing factors for the prognostic difference.

**Results:**

There were 805 resectable patients for the final analysis. Diffuse-type comprised of 48.7% in the gastric carcinoma in our group and showed poorer prognosis than intestinal-type (P=0.013). Multivariate analysis revealed that independent prognostic factors for gastric carcinoma patients were T stage (P<0.001), N stage (P<0.001) tumor size (P<0.001) and Lauren classification (P=0.003). For the clinical features, diffuse-type was significantly associated with younger age (p<0.001), female preponderance (p <0.001), distal location (P<0.001), advanced pT (p < 0.001), advanced pN (p < 0.001) and advanced TNM stage (p = 0.027).

**Conclusions:**

Diffuse type adenocarcinoma carries a worse prognosis that may be partially explained by the tendency of this subtype to present at more advanced T and N stage. However, Lauren classification has prognostic significance that is independent of T and N stage as well as other prognostic variables based on the multivariate cox analysis.

## Background

About one million people are diagnosed with gastric carcinoma each year all over the world, making it the fourth most common cancer and the second leading cause of cancer related death [1]. The incidence rate of gastric carcinoma varies dramatically from one part of the world to another and it is particularly common in Eastern Asia, including China [2]. The prognosis for gastric adenocarcinoma patients remains poor and our understanding of this cancer entity is still limited.

According to the Lauren classification, gastric adenocarcinomas can be divided into two major histological types, diffuse and intestinal type [3]. The intestinal type is characterized by cohesive cells which form gland-like structures, while for the diffuse type, tumor cells lack cell-to-cell interactions and infiltrate the stroma as single cell or small subgroups, leading to a population of non-cohesive, scattered tumor cells [3]. Although the Lauren classification system can date back to 1965, it is still widely accepted and employed by pathologists and physicians today and represents a simple but robust classification approach. The two Lauren types have several distinct clinical and molecular characteristics, including etiology, carcinogenesis, epidemiology and progression, message ribonucleic acid (mRNA) and / or protein expression profile, microsatellite instability, and mutation profiles [4]. Thus, it is widely accepted that they represent distinct disease entities which may benefit from different therapeutic approaches. In the recent reported clinical trial—Trastuzumab in combination with chemotherapy versus chemotherapy alone for treatment of HER2-positive advanced gastric or gastro-oesophageal junction cancer (ToGA), patients in the control group had a higher overall survival rates than expected [5] and the authors considered that it might be due to the higher percentage of intestinal-type tumors in the control group compared with other phase III studies [6]. It was reported that the expression of human epidermal growth factor receptor-2 (HER2) was more common in intestinal-type tumors and such patients have a better outcome than patients with diffuse-type tumors [7-10]. The importance of Lauren classification took our attention.

The aims of our present study are: (1) to analyze the prognostic value of Lauren classifications in resectable gastric cancer patients in China, (2) to compare the clinicopathological characteristics of diffuse-type and intestinal-type in gastric cancer and identify the clinicopathological factors which may explain the different prognosis of the these two types.

## Materials and methods

### Ethics statement

All patients provided written informed consent for their information to be stored and used in the hospital database. Study approval was obtained from independent ethics committees at Cancer Center of Sun Yat-Sen University. The study was undertaken in accordance with the ethical standards of the World Medical Association Declaration of Helsinki.

### Patients

The medical records of 1000 patients with pathologically-confirmed gastric adenocarcinoma between January 1996 and December 2006 were retrospectively analyzed. They all received D2 resection carried out by experienced surgeons in the Cancer Center of Sun Yat-Sen University following the Japanese Gastric Cancer Association (JGCA) guidelines [11]. Both the proximal and distal margins are negative and at least 3 cm away from the tumor. Besides, the surgeon dissected the station D2 lymph nodes. There is no macroscopic or microscopic residual tumor. The total number of dissected lymph nodes of the 1000 gastric carcinoma patients was 16008, with an average of 18.8 ±5.3 (means±s.d.) dissected nodes per case (median 24.0, range 13–72). The number of excised lymph nodes was less than 15 in 24.2% of patients who received resection.

We excluded 82 patients (8.2%) because of missing baseline characteristics, 75 patients (7.5%) younger than 18 years old, 32 patients (3.2%) with incomplete follow-up and 6 patients (0.6%) with secondary malignancy. None of the patients received neoadjuvant treatment. The final study involved 805 patients.

The clinical features collected for subsequent analysis included gender (male or female), age at diagnosis (<59 or ≥59 years, the median age was 59), tumor size (≤5 cm or >5 cm, the median diameter was 5 cm), location of primary tumor (proximal or distal), histology subtypes (well + moderate differentiated adenocarcinoma or poorly + signet ring cell differentiated adenocarcinoma), Lauren classifications (diffuse type or intestinal type), anemia (yes or no), angiolymphatic invasion (yes or no), the TNM staging system (American Joint Committee on Cancer (AJCC) 7^th^ edition) (Table 1).

**Table 1:** Demographics and univariate survival analysis results of the 805 gastric carcinoma patients

**Factors**	**Numbers**	**5 year survival rate (%)**	**P value**
Gender			
Male	557	48.9	
female	248	46.1	0.581
Age median 59			
≤59	419	50.5	
>59	386	45.8	0.135
Lauren classification			
Diffuse type	392	44.1	
Intestinal type	356	52.7	0.013
Tumor size			
≤5 cm	483	55.2	
>5 cm	322	38.5	<0.001
Anemia			
Yes	247	57.4	
No	558	61.0	0.554
Angiolymphatic invasion			
Yes	52	36.5	
No	753	48.8	0.009
Location of tumor			
Proximal	347	42.5	
Distal	458	49.4	0.017
Number of lymph nodes			
<15	195	46.9	
≥15	610	51.4	0.233
Type of gastrectomy			
Proximal subtotal	323	45.2	
Distal subtotal	386	56.9	
Total	96	31.5	<0.001
The 7^th^ T stage (AJCC)			
T1	59	88.1	
T2	87	67.8	
T3	532	45.3	
T4	127	27.5	<0.001
The 7^th^ N stage (AJCC)			
N0	269	69.0	
N1	125	59.6	
N2	179	44.0	
N3	232	23.0	<0.001
The 7^th^ TNM stage (AJCC)			
IA	56	86.3	
IB	70	76.9	
IIA	96	70.7	
IIB	206	66.2	
IIIA	82	59.5	
IIIB	132	43.7	
IIIC	163	24.3	<0.001

Abbreviations: *AJCC*, American Joint Committee on Cancer; *TNM*, Tumor-Node-Metastasis.

During the study period we did not have a standardized protocol for postoperative chemotherapy and (or) radiotherapy. Adjuvant therapy was considered in patients with T3-T4 classification and/or positive lymph node involvement. In the present study, only 532 (66.1%) patients completed the adjuvant chemotherapy (2–6 cycles). Agents using for chemotherapy included oxaliplatin, 5-fluorouracil, capecitabine, S-1, irinotecan, docetaxol and taxol. The median number of cycles was 4. No patients received adjuvant radiotherapy. As of May 1^st^, 2012, 403 patients had died from the disease.

Tissue samples from resected tumors were classified and staged by an experienced pathologist according to the WHO classification and the TNM staging system following general pathological guidelines. Assignment of the histologic type was based on the Lauren criteria. The intestinal type was described as a tumor with glandular architecture, resembling colonic carcinoma; the diffuse type, as a tumor composed of solitary or small clusters of cells, and lacking glandular structures. The mixed type was described as the combination of these two features. Two pathologists reviewed the original diagnostic slides in order to grade, stage and classify the tumors as intestinal or diffuse type.

### Statistical analysis

All statistical analyses were performed by Statistical Package of Social Sciences 13.0 software. P value < 0.05 was considered to be statistically significant. The Kaplan-Meier method was used to estimate overall survival. For patients who remained alive, data were censored at the date of the last contact. Kaplan-Meier analysis with log-rank testing was used for univariate analysis. The definition of the overall survival interval was the duration between the date of diagnosis and the date of last contact. Variables showing a trend for association with survival (P < 0.05) and variables that were known to have prognostic value were selected in the final multivariable Cox proportional hazards model, while variables that are highly associated with others were excluded from the final multivariable model. The chi-square test was used to compare the clinicopathologic data.

## Results

### Patient demographics

The median age of the 805 patients was 59 years (ranging from 20 to 84 years old). Among them 557 were male and 248 were female. The overall 5-year survival for the patient population was 48.2%, with a median survival of 53.4 months. The median follow-up for the entire cohort was 42.0 months (range 3.0–173.0 months). The characteristics of the 805 gastric adenocarcinoma patients and the effect of clinical features on survival are summarized in Table 1.

### Lauren classification

There were 396 (49.2%) patients with diffuse-type and 352 (43.7%) patients with intestinal-type carcinoma. The remaining 57 patients belonged to the mixed-type carcinoma. In the following analysis, we only included the 748 patients with the diffuse-type or intestinal-type carcinoma.

Patient characteristics of the two groups are shown in Table 2. Among intestinal-type carcinoma, 146 (41.5%) were younger than 60 years old compared to 246 (62.1%) of patients with diffuse-type carcinoma (P<0.001). The ratio of male to female was significantly higher in the intestinal-type carcinoma group than that in the diffuse-type group, 3.1 vs 0.86 (P<0.001). We divided the ten year study period into two groups, the early period: from January 1996 to December 2000, the later period: from January 2001 to December 2006. We found that the ratio between diffuse type to intestinal type decreased from 1.57 (early period) to 1.03 (later period), P=0.041. Among intestinal-type carcinomas, 55.3% of tumors were located in the proximal stomach compared to 32.9% of diffuse-type carcinomas, (P <0.001). The mean size of tumors was about 51.6 mm for diffuse-type carcinomas and 50.2 mm for intestinal-type , P=0.268. Distribution of T-stage was significantly different between the diffuse-type and the intestinal-type (Table 2). Moreover, for the lymph nodes status, 41.3% of patients with intestinal-type carcinoma had no evidence of lymph nodes metastasis, while for the diffuse-type carcinoma, the rate of N0 was 28.6%. Besides, the ratio of stage I: II: III was 12.1%:37.2%: 50.7% in the diffuse-type, nevertheless, it was 18.5%: 37.7%: 43.8% in the intestinal-type, P=0.027.

**Table 2:** Clinicopathologic features and Lauren classification

**Characteristics**	**Total number**	**Lauren classification**		**P value**
		**Diffuse type**	**Intestinal type**	
Age				<0.001
≤59	392	246	146	
>59	356	150	206	
Sex				<0.001
Female	298	211	87	
Male	450	181	269	
Time period				0.041
1996.1–2000.12	118	72	46	
2001.1–2006.12	630	320	310	
Location of tumor				<0.001
Proximal	326	129	197	
distal	422	263	159	
Size				0.268
≤5 cm	453	230	223	
>5 cm	295	162	133	
The 7^th^ T stage (AJCC)				<0.001
T1	61	24	37	
T2	79	55	24	
T3	532	260	272	
T4	76	57	19	
The 7^th^ N stage (AJCC)				<0.001
N0	259	112	147	
N1	116	52	64	
N2	161	92	69	
N3	212	140	72	
The 7^th^ TNM stage (AJCC)				0.027
I	113	47	66	
II	280	146	134	
III	355	199	156	
Histology subtype				<0.001
Well + Moderate	281	0	281	
Poor + signet ring cell	467	392	75	

Abbreviations: *AJCC*, American Joint Committee on Cancer; *TNM*, Tumor-Node-Metastasis; The analysis only includes 748 patients, and excludes 57 mixed diffuse-intestinal types.

### Univariate and multivariable analyses of overall survival

**Figure 1:**
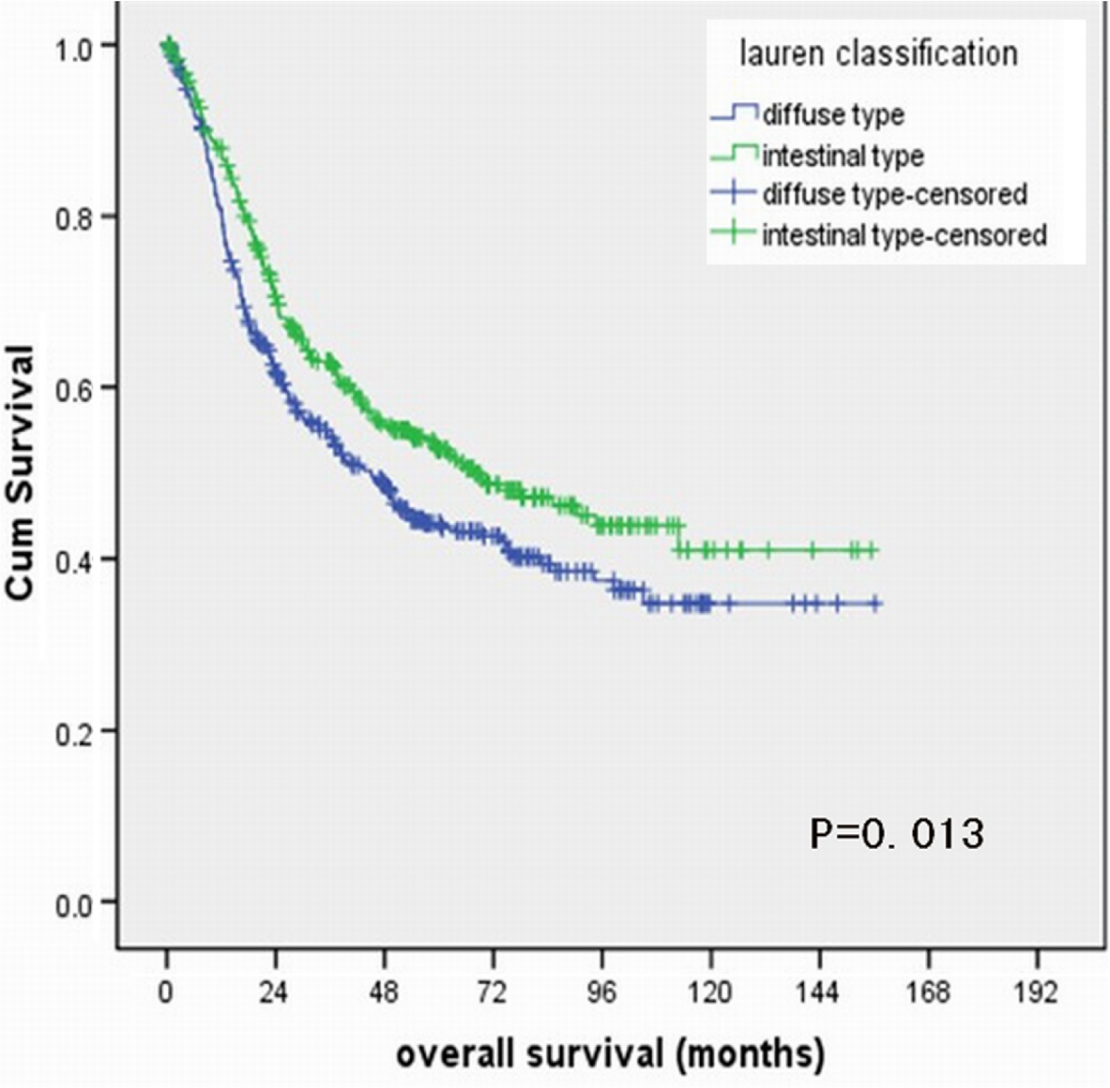
Survival curves of gastric adenocarcinoma patients with diffuse-type and intestinal-type.

Both univariate and multivariable analyses were used to evaluate factors relating to overall survival. Factors of Lauren classification, tumor size, angiolymphatic invasion, location of tumor, degree of differentiation, T stage, N stage and TNM stage in the AJCC 7^th^ system were significantly associated with overall survival (Table 1). The 5-year overall survival rate of patients with diffuse-type and intestinal type were 44.1% and 52.7%, respectively, P=0.013 (Figure 1).

For the multivariable regression analysis, only T stage (P<0.001), N stage (P<0.001),tumor size (P<0.001) and Lauren classification (P=0.003) remained independent negative predictors of survival (Table 3).

**Table 3:** Multivariate analysis of overall survival in gastric carcinoma

**Factors**	** Characteristics**		**Hazard ratio**	**95%CI**	**P value**
	**Unfavorable**	**Favorable**			
Age	≥59	<59	1.111	0.899–1.374	0.328
Gender	Female	Male	1.057	0.843–1.324	0.631
Lauren classification	Diffuse type	Intestinal type	0.736	0.595–0.910	0.005
Histological grade	Poorly	Well/moderately	1.025	0.899–1.167	0.714
Size	>5 cm	≤5 cm	1.605	1.307–1.972	<0.001
Location	Proximal	Distal	0.852	0.697–1.042	0.120
T stage	2/3/4	1	1.890	1.230–2.746	<0.001
N stage	1/2/3	0	1.368	1.048–1.894	<0.001
Angiolymphatic invasion	(+)	(−)	1.209	0.831–1.759	0.321
Type of gastrectomy	Total	Subtotal	1.135	0.859–1.468	0.582

*CI*, confidence interval.

## Discussion

In 1965, Lauren proposed a division of gastric carcinoma into two structurally distinct types, the intestinal-type and the diffuse-type [3]. The former was most frequently seen in men and older patients and the latter had a worse prognosis and was more frequent in women and younger patients. He proposed that these two types could account for approximately 85% of gastric carcinomas, and the remainder comprised mixed types and other less common histologies. Several studies have found that the Lauren classification is reproducible [12-16]. Our study confirmed the fact that patients with the diffuse-type of gastric carcinoma had poorer prognosis than patients with intestinal type (P = 0.013) in our Chinese patient population. Multivariate analysis revealed that independent prognostic factors for patients with resected gastric adenocarcinoma were TNM stage, tumor size and the Lauren classification. Our previous studies also showed that tumor size was an independent negative predictor of survival in lymph nodes negative gastric carcinoma patients [17]. While the histology was eliminated. Several studies have indicated that patients with intestinal-type tumors had a better outcome than those with diffuse-type tumors [8-10]. The two Lauren types varied in several clinical and molecular characteristics. We tried to seek the difference between these two types in the clinical features which may be responsible for the survival variation.

In our study, diffuse-type gastric carcinoma was significantly associated with younger age (p <0.001), female preponderance (p <0.001), distal location (P<0.001), advanced pT (p < 0.001), advanced pN (p < 0.001) and advanced TNM stage (p = 0.027). Though the impact of age, gender and location as prognostic factors was still controversial, it is well accepted that advanced pN as well as advanced pT are poor prognostic factors for gastric carcinoma [17-20]. The higher percentage of patients with advanced pN and advanced pT subtypes in the diffuse-type gastric carcinoma as compared with the intestinal-type may contribute to the poor prognosis of patients with diffuse-type. Yamashita K et al. revealed that the more dismal prognosis of diffuse-type gastric carcinoma than intestinal-type could be explained by propensity of deeper invasion and emerging peritoneal cancer cell in the Japanese population [8]. In our study, we demonstrated that high percentage of advanced pN was also responsible for the poor prognosis of diffuse-type in Chinese patients with resectable gastric carcinoma.

The present study shows that both diffuse-type and intestinal-type carcinoma account for 92.9% (748/805) of gastric carcinoma in China. Diffuse-type accounted for 48.7% (392/805) of all gastric carcinomas. It was reported that intestinal type was more common in areas with a high risk for gastric carcinoma, whereas diffuse type was relatively more common in low-risk areas [13]. The ratio of diffuse and intestinal types of gastric carcinoma in China (1.13) was similar to the Hawaii Japanese population, which was 1.32, while in European countries, such as Finland and Portugal, intestinal type was more common (Table 4) [13,16,21,22].

**Table 4:** Ratio of the diffuse type to intestinal type of gastric carcinoma in different areas

**Area**	**Diffuse type**	**Intestinal type**	**Mixed type**	Ratio*
Our data	396	352	57	1.13
Hawaii Japanese ^16^	144	109	41	1.32
Singapore ^21^	206	405	37	0.51
Portugal ^22^	48	112	12	0.43
Finland ^25^	537	729	177	0.74

* Ratio means the ratio of the diffuse type to intestinal type gastric carcinoma.

In our study we found that the intestinal-type was more frequently in males and in elderly patients, while the diffuse-type occurred more frequently in women and young patients. This results confirms the observations of others [12-15]. The 3.6 ma1e:female ratio for the intestinal type and 0.86 ratio for diffuse tumors in our study is comparable to that noted in Fukuoka, Japan , where they were 2.60 and 0.5 respectively [23], and in Hawaii Japanese, where these ratio were 2.39 and 0.86 respectively [18].

In China, not all the patients would go to the same hospital during the course of treatment, maybe after recurrence they would transfer to the local hospital or quit treatment. We tried to get the data of recurrence but some of the patients forgot the date of recurrence. That is why we don’t analyze the recurrence free survival.

In our previous study, we showed that as the age increased there was a steady increasing in the proportion of male and steady decreasing of female [24]. Similarly, we presently also found a steady increasing in the ratio between male and female as the age increased. This suggests a positive relationship between the age and the male: female ratio, however the exact nature of the correlation between gender, age and Lauren classifications warrants further study.

In our study, we found a decrease in the ratio between diffuse type to intestinal type over time from 1.57 (from January 1996 to December 2000) to 1.03 (from January 2001 to December 2006), P=0.041. However, Pekka A et al. found that in a Finland based population,the ratio of diffuse-type to intestinal-type increased over time suggesting differences in the epidemiologic shift of gastric carcinomas between Eastern and Western populations [25].

The limitation of current study is its retrospective methodology from a single-institution experience. The impact of various treatments related outcome could not be fully evaluated. Moreover, it is inaccurate to analyze the recurrence free interval. External validation by using other large database for evaluating the prognostic effect of Lauren classification would be of value to further explore the mechanism of different prognosis between diffuse-type and intestinal-type gastric carcinoma.

## Conclusion

The authors are not aware of any previous studies which address the clinicopathological characteristics and prognostic impact of the Lauren classification in patients with resectable gastric cancer in China. In this retrospective study conducted with 805 patients with gastric adenocarcinoma we submit the following conclusions: 1) The combination of TNM staging with Lauren classification and tumor size are the most meaningful prognostic factors. 2) Advanced pN as well as advanced pT appear to account for the poor prognosis of diffuse-type in Chinese patients with resectable gastric carcinoma.

## Abbreviations

mRNA: Messenger ribonucleic acid; ToGA: Trastuzumab in combination with chemotherapy versus chemotherapy alone for treatment of HER2-positive advanced gastric or gastro-oesophageal junction cancer; HER2: Human epidermal growth factor receptor-2; JGCA: Japanese gastric cancer association; AJCC: American joint committee on Cancer; WHO: World health organization; TNM: Tumor-node-metastasis.

## Competing interests

We have no financial or personal relationships with other people or organizations that would bias our work. No benefits in any form have been received or will be received from a commercial party related directly or indirectly to the subject of our article. We declare that we have no competing interests.

## Authors’ contributions

MZQ participated in the clinical data collecting of the gastric carcinoma patients and drafted the manuscript. MYC and MZQ reviewed the pathologic slices and decided the Lauren classification. DSZ performed the statistical analysis. ZQW participated in the design of the study. DSW and YHL participated in the statistical analysis. RHX conceived of the study, and participated in its design and coordination and helped to draft the manuscript. All authors read and approved the final manuscript.

## Authors’ information

Miao-zhen Qiu and Mu-yan Cai are co-first authors.
